# Nigella Sativa and Thymoquinone for Prevention or Mitigation of Acquired Sensorineural Hearing Loss: A Systematic Review

**DOI:** 10.3390/jcm14238433

**Published:** 2025-11-27

**Authors:** Hunor Levente Horvath, Violeta Necula, Maximilian George Dindelegan, Cristina Maria Blebea, Victor Esanu, Alma Aurelia Maniu

**Affiliations:** 1 Department of Otorhinolaringology, “Iuliu Hatieganu” University of Medicine and Pharmacy, 4–6 Clinicilor Street, 400006 Cluj-Napoca, Romania; hunorlevente.horvath@gmail.com (H.L.H.); cristina_blebea@yahoo.com (C.M.B.); almacjro@yahoo.com (A.A.M.); 2 Institute of Oncology “Prof Dr. Ion Chiricuta”, 34–36 Republicii Street, 400015 Cluj-Napoca, Romania; maximilian.dindelegan@gmail.com; 3 Department of Surgery—Practical Abilities, “Iuliu Hatieganu” University of Medicine and Pharmacy, 23 Gheorghe Marinescu Street, 400012 Cluj-Napoca, Romania; 4 Center for Complex Microvascular Surgery, Erasmus MC, 40, Na-2112 Dr. Molewaterplein Street, 3015 GD Rotterdam, The Netherlands; victor.esanu@elearn.umfcluj.ro; 5 Department of 1st Surgery, “Iuliu Hatieganu” University of Medicine and Pharmacy, 3–5 Clinicilor Street, 400006 Cluj-Napoca, Romania

**Keywords:** sensorineural hearing loss, ototoxicity, Nigella sativa, thymoquinone

## Abstract

**Background/Objectives:** Acquired sensorineural hearing loss (SNHL) can result from a wide range of insults, including ototoxic drugs, Meniere’s disease, noise-induced ototoxicity, and aging. The underlying pathophysiological mechanism arises through damage to the inner ear via oxidative stress and inflammation. Recent research suggests that natural antioxidants are promising solutions to prevent SNHL. Nigella sativa (NS), through its active compound thymoquinone (TQ), is a potent antioxidant that has shown promising results. The aim of this systematic review is to examine whether NS can offer protection against acquired SNHL. **Methods**: This study reviewed the literature on the protective effects of Nigella sativa oil (NSO) or TQ against acquired SNHL. We followed the guidelines of the Preferred Reporting Items for Systematic Reviews and Meta-Analyses (PRISMA). A comprehensive literature search was conducted across multiple databases using keywords related to NS and hearing loss. Meta-analyses were performed on eligible studies. Risk of bias was assessed using the Systematic Review Center for Laboratory Animal Experiments (SYRCLE) tool for animal studies. **Results**: Out of a total of 76 records, 38 duplicates were removed. From the remaining 38, 13 studies met the inclusion criteria. Multiple studies reported a significant protective effect of NS, especially against ototoxicity. The risk of bias across the studies was moderate. **Conclusions**: Preclinical evidence indicates that NS provides significant protection against acquired SNHL. These protective effects are attributed to their antioxidant, anti-apoptotic, and anti-inflammatory properties. Overall, our systematic review highlights NS as a promising candidate for preventing SNHL.

## 1. Introduction

Sensorineural hearing loss (SNHL) is a common condition resulting from injury to the inner ear or auditory nerve that may be congenital or acquired, in most cases being irreversible. Acquired SNHL can have multiple etiologies, such as autoimmune diseases, infections, excessive noise exposure, age-related degeneration, or iatrogenic hearing loss due to ototoxic medication [1,2].

Ototoxicity refers to the capacity of certain chemicals to impair function and damage the sensory and supporting cells of the inner ear. Common ototoxic agents include salicylates, loop diuretics, aminoglycosides, anti-malarial drugs, heavy metals, and platinum-based chemotherapeutic agents [3]. One of the most frequent causes of SNHL due to ototoxicity is platinum-based chemotherapy used in cancer treatment [4].

Platinum-based chemotherapy-induced hearing loss affects around 500,000 people annually worldwide [4]. Cisplatin is the most commonly used platinum-based chemotherapy agent in the treatment of various malignancies, such as testicular, ovarian, lung, breast, and head and neck cancers [4,5]. However, due to its non-specific mechanism of action, it affects both cancer cells and healthy tissues. Hearing loss is dose-dependent, bilateral, symmetric, progressive, and irreversible, beginning at high frequencies. The rate of cisplatin-induced SNHL varies widely in the literature, ranging from 11% to 97%, with an average incidence of 62% [6]. Aminoglycosides are another major class of ototoxic drugs. The resulting hearing loss primarily affects high frequencies, often continues to progress even after treatment has ended, and is generally regarded as permanent [7].

Noise-induced hearing loss (NIHL) arises with sustained exposure to levels exceeding ~85 dB, damaging outer hair cells and supporting cells of the organ of Corti [8]. Age-related hearing loss (ARHL), or presbycusis, arises from the cumulative impact of aging on the auditory system. It is a gradual, bilateral, high-frequency SNHL [9].

Accumulating evidence implicates oxidative stress and reactive oxygen species (ROS)—driven molecular damage represents pivotal mechanisms in the pathogenesis of acquired SNHL, including ARHL, NIHL, and ototoxicity [10,11,12].

Although there are currently no established treatments for acquired SNHL, some therapies have shown promise. Corticosteroids, such as dexamethasone, are frequently used in cases of sudden idiopathic sensorineural hearing loss or inner ear inflammation due to their anti-inflammatory effects [13]. They modulate the activity of antioxidant enzymes, inhibit the synthesis of pro-inflammatory molecules, and reduce cellular apoptosis. However, their effectiveness can vary, and systemic administration can lead to important side effects [14,15]. Recent studies have emphasized ROS-targeted interventions and antioxidant strategies as promising avenues for the prevention and treatment of acquired SNHL [12,16]. Sodium thiosulfate (STS), a strong nucleophile, has proven effective in binding and neutralizing cisplatin, thus preventing its ototoxic effects [17,18,19]. The US Food and Drug Administration and the European Medicines Agency recently approved the administration of STS in children over 1 month treated with cisplatin as an otoprotectant. Recent studies demonstrated that systematic STS safely reduces the incidence and severity of hearing loss in children with localized disease when administered within a 6 h window [20,21].

Nigella sativa (NS) belongs to the Ranunculaceae family and is found in the Middle East and Southwest Asia. It is an herbaceous plant known for its various therapeutic effects, including anti-inflammatory, antibacterial, antioxidant, and immunomodulatory properties [22,23,24,25]. Its major bioactive constituent, thymoquinone (2-methyl-5-isopropyl-1,4-benzoquinone), is considered the principal molecule of the seed oil, is a quinone known for scavenging free radicals and protecting tissues from oxidative damage, and is widely used as a purified surrogate for NS [26]. Thymoquinone plays a key role in reducing the generation of ROS by inhibiting the activity of xanthine oxidase. Moreover, it serves as a potent antioxidant by neutralizing free radicals, binding metals to control cellular oxidation processes, modulating stress-related enzymes, and boosting endogenous antioxidant production [27,28,29].

In hearing research, NS and/or its active constituent TQ have shown potential to reduce oxidative stress and inflammation in the inner ear [30]. This prompts the question of whether NS/TQ could protect the inner ear from harm caused by ototoxic agents, trauma, or age-related hearing loss. While initial findings are encouraging, there has not yet been a thorough synthesis of this research.

Therefore, we conducted a systematic review of all available studies in the scientific literature to evaluate the potential role of NS/TQ in either preventing or reducing SNHL. Our study aimed to systematically summarize the evidence and assess its quality, thereby providing clarity on whether Nigella sativa truly holds a promise in the fight against acquired SNHL.

## 2. Materials and Methods

This systematic review was designed following the Preferred Reporting Items for Systematic Reviews and Meta-Analyses (PRISMA) 2020 guidelines (Supplementary Materials) [31]. Although the protocol for this review was written prior to study selection and data extraction, it was not prospectively registered. To ensure transparency and reproducibility, the complete protocol has been retrospectively registered and made publicly available on OSF (DOI: 10.17605/OSF.IO/M49UF).

Eligibility Criteria

We included preclinical studies where the intervention involved Nigella sativa (in any form—seed extract or oil) and/or its main active compound, Thymoquinone, administered systemically (oral gavage, intravenous, or intraperitoneal) or via a local route (intratympanic injection) and that evaluated its effect in either preventing or treating acquired sensorineural hearing loss (iatrogenic, noise-induced, age-related, infectious, or autoimmune disease-related). Primary outcomes were quantitative measures of hearing function; secondary outcomes included histologic evidence of cochlear protection and any adverse effects/toxicity. The review included preclinical studies, encompassing animal models or cell cultures, subjects of any age or sex, and the comparison was either a placebo/control group or an established treatment. Human clinical studies were identified but excluded from the main synthesis; these are mentioned only in the Discussion section. The studies included were interventional designs (e.g., randomized controlled trials and other experimental models) with clearly described methodology and results, published in English, with no period restrictions.

Exclusion criteria were studies focusing on conductive hearing loss; non-interventional studies (purely observational, reviews); conference abstracts without extractable data; mechanistic studies lacking hearing outcomes; studies without clear methodology or quantifiable hearing-related outcomes in animal and human studies; and non-English papers.

Information Sources and Search Strategy

We performed a comprehensive literature search across PubMed/MEDLINE, Web of Science, Scopus, and Embase from inception to 3 August 2025, to ensure maximal coverage. Reference lists of all included papers and relevant reviews were hand-searched to find additional studies.

The search combined terms related to Nigella Sativa/TQ, hearing loss, and auditory protection, and we used both scientific and common names. The keywords were then used to generate a comprehensive search string (Appendix A).

Study selection and data collection process

All identified papers from the initial search were imported into a reference manager (Covidence), and all duplicates were removed. Two reviewers (HHL and VE) independently screened titles/abstracts, then full text, according to the inclusion and exclusion criteria. Any disagreements during the screening process were resolved through discussion, and when necessary, by consultation with a third reviewer (AAM).

A standardized extraction form captured publication details; study design; sample size; intervention (substance, dose, route, timing relative to insult, duration, comparator); hearing-loss model; outcome measures and key results; and adverse effects/toxicity. Data were collated in a structured spreadsheet and presented in a table. Because the included studies varied in species, ototoxicity model, timing and route of intervention, and auditory outcome measures, we used a dual-approach synthesis. All studies were summarized descriptively in structured tables, while studies reporting extractable numerical outcomes that were eligible were included in a random-effects meta-analysis.

Risk of Bias Assessment

Two reviewers (HHL &VE) assessed the risk of bias using study-appropriate tools: the SYRCLE tool for animal studies [32,33]. Each domain assessed according to the tools’ criteria was rated as high, low, or unclear risk. Furthermore, we noted any specific methodological strengths or limitations that could influence the results.

Outcome Measures

To objectively assess the protective effects of NS, the primary outcome was preservation or improvement of hearing based on quantitative metrics. In the animal studies, this was evaluated by changes in auditory brainstem response (ABR) thresholds and/or Distortion product otoacoustic emission (DPOAE) amplitudes, comparing the study group to the control group. Some animal studies also performed cochlear histopathology, which was considered supportive evidence of hearing protection. Secondary outcomes included any adverse effects or toxicity related to the administration of NS/TQ.

Statistical Analysis

All statistical analyses were performed in R (version 4.3.2) using the metafor package. Effect sizes were calculated for all outcomes with available means, standard deviations, and sample sizes, using Hedges’ g standardized mean differences (SMDH). ABR thresholds were direction-corrected so that positive values consistently reflected a protective effect. A random-effects model (REML) was applied due to anticipated heterogeneity across ototoxicity models, dosing regimens, and outcome measures. Heterogeneity was assessed using τ^2^, I^2^, and Cochran’s Q, and a 95% prediction interval was computed.

Multi-arm studies using the same animal cohort were treated as separate comparisons but accounted for using a multilevel model with random intercepts for study. Frequency-specific DPOAE outcomes from the same animals were pooled into a single effect size according to Cochrane recommendations.

Subgroup analyses were conducted for the ototoxicity model, compound, and outcome type. Publication bias was evaluated using funnel plots and Egger’s test, and sensitivity analyses included leave-one-out recalculations and influence diagnostics.

## 3. Results

### 3.1. Study Selection

**Figure 1:**
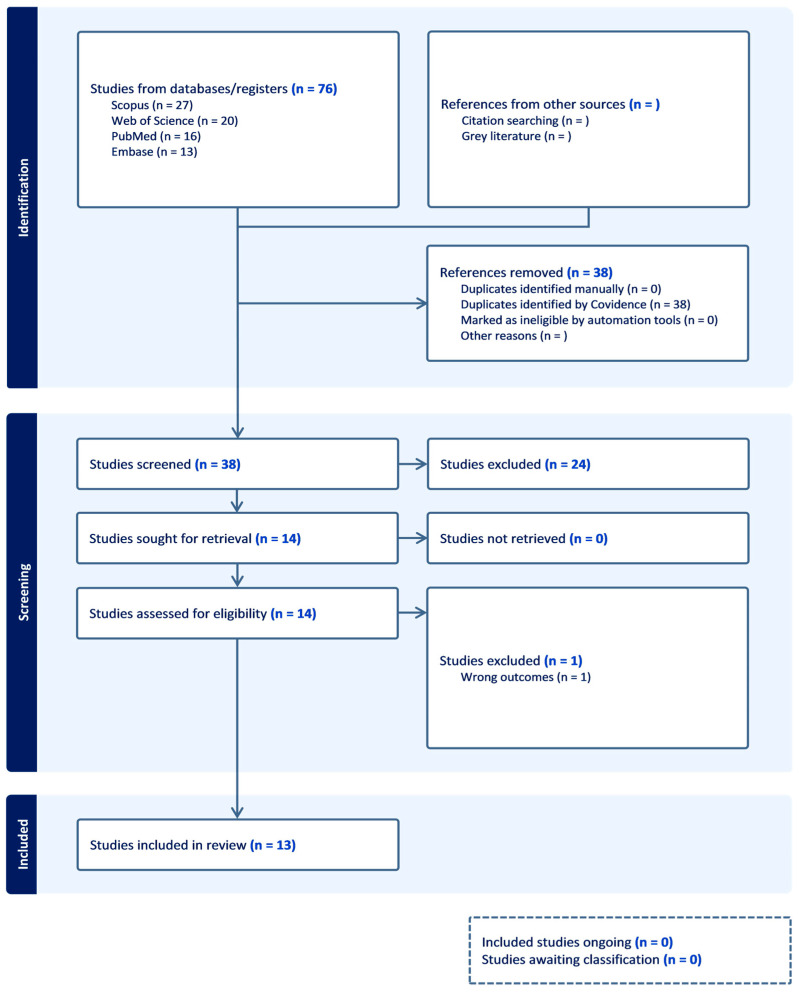
PRISMA flowchart exhibiting the study selection process.

The initial search yielded 76 articles; 38 duplicates were removed, leaving 38 for screening. Of these, twenty-three were excluded at title/abstract screening due to unrelated topics, lack of focus on NS/TQ or hearing outcomes, letters, or reviews. Subsequently, fifteen studies underwent full-text reading. One article was excluded at this stage for not assessing hearing outcomes or ototoxicity. Ultimately, 13 studies met the eligibility criteria and were included in the review (Figure 1): 12 in vivo animal studies and 1 in vitro, cell-culture study.

### 3.2. Study Characteristics

Animal studies, published between 2013 and 2024, were conducted on rodents; of these, 10 were performed on rats, one used mice, and one employed guinea pigs. Sample sizes ranged from 16 to 48, with an average of 5–10 animals per group. The in vitro study was performed on mouse cochlear hair cell cultures.

The experimental models covered a range of common causes of acquired SNHL. Oto-toxicity was the most frequently modeled condition: two studies used cisplatin, and six used aminoglycoside antibiotics. Four of the studies used a noise-induced hearing loss model, and one used the age-related hearing loss model in mice.

Nigella Sativa was administered either as Nigella sativa oil (NSO) extract or as purified TQ. Delivery methods included oral gavage and intraperitoneal injection, with one study using intratympanic injection. Timing of administration varied; administration protocols were prophylactic, therapeutic, or combined. Control groups were usually a placebo or no treatment, and some studies also included an NSO/TQ-only group to identify any potential toxic effects.

All animal models assessed hearing using quantitative measurements, by using ABR, DPOAE, or both, and one study used a behavioral audiogram. Four studies also performed histology, and biochemical assays were performed in one case. Follow-up durations were short-term (days to weeks), except for the ARHL model (1 month).

### 3.3. Risk of Bias

**Figure 2:**
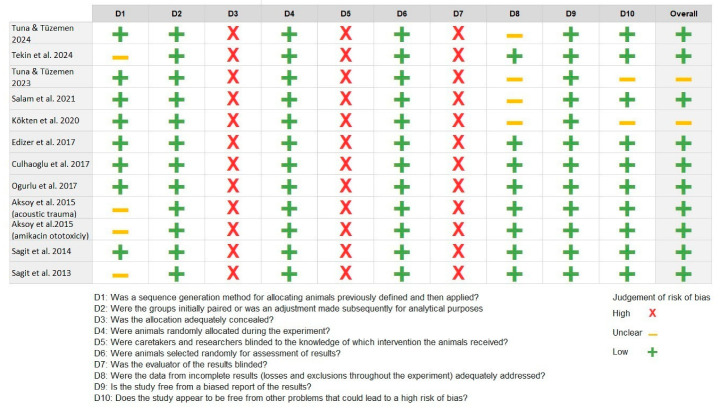
Risk of bias using the Systematic Review Center for Laboratory Animal Experiments [34,35,36,37,38,39,40,42,43,44,45,46].

Using the SYRCLE tool, most animal studies were rated as having low to unclear risk of bias, with full results presented in Figure 2.

### 3.4. Results of Individual Studies

Because Nigella sativa and its active constituent thymoquinone (NS/TQ) act through overlapping antioxidant and anti-inflammatory mechanisms and were used in similar experimental settings, the results are presented grouped by the type of cochlear insult (drug-induced, noise-induced, age-related), rather than by compound. Within each model, we specify whether the intervention consisted of NSO or isolated TQ.

#### 3.4.1. Drug-Induced Hearing Loss

Cisplatin-induced ototoxicity

Sagit et al. administered rats TQ intraperitoneally (i.p.) at 40 mg/kg for 5 days (beginning two days before cisplatin administration). ABR thresholds showed statistically significant differences in post-treatment values between the treatment and no-treatment groups (49.50 ± 19.86 vs. 21.11 ± 6.76). DPOAE values in the cisplatin group were significantly decreased compared to the TQ-treated group. No TQ-related toxicity observed [34].

In a study performed by Kokten et al., rats were administered cisplatin i.p. for 5 days (days 1, 3, and 5) and NSO was given through oral gavage, 3 mL/day for 5 days (days 1, 3, 5, 7, 9). ABR thresholds were significantly different between the cisplatin-only and cisplatin + NSO group at 16 (43 ± 17.02 vs. 26.5 ± 4.11) and 32 kHz frequencies (55.5 ± 17.70 vs. 25 ± 4.71), with histology showing reduced cochlear damage. One death occurred in each cisplatin group; no NSO-related mortality was observed [35].

Aminoglycoside-induced ototoxicity

In another study by Sagit et al., gentamicin was administered i.p. with or without TQ (20 mg/kg daily for 15 days). ABR showed significantly lower thresholds in the TQ-treated group compared to the gentamicin-only group (16.67 ± 6.37 vs. 45.83 ± 11.38). Moreover, immunohistochemistry revealed lower inner and outer hair cell damage in the TQ-treated group [36].

Tuna et al. conducted a dose-ranging study evaluating the effects of NSO administered i.p. in gentamicin ototoxicity. The results showed a significant difference when pretreatment and post-treatment measurements were compared between the gentamicin + saline group and the gentamicin + 0.3 mL/kg NSO group (51.25 ± 12.99 vs. 12.5 ± 3.98). There was no significant difference between the 0.1 mL/kg and 3 mL/kg NSO groups. Furthermore, post-treatment ABR results did not differ significantly between the prophylactic and therapeutic groups for the 0.3 mL/kg group (12.5 ± 3.98 vs. 10 ± 0) [37].

In a follow-up study, Tuna et al. investigated the therapeutic effect of delayed administration of NSO. Rats received 0.3 mL/kg NSO i.p. daily for 5 days, starting from either day 10 or 25 after initiating gentamicin. There was no statistically significant difference in ABR thresholds between the early and late-administration groups (10 vs. 10.3 ± 2.88) [38].

Edizer et al. administered guinea pigs NSO through intratympanic injections. Post-treatment ABR results showed a significant difference between gentamicin-only and gentamicin + NSO at click stimuli (61.4 ± 11.0 vs. 47.9 ± 8.9). Hearing outcomes were different at 8 kHz tone-burst stimuli as well (58.6 ± 11.0 vs. 50.7 ± 12.7); however, the difference did not reach statistical significance. Histology revealed preserved cochlear nerve structures in the NSO group compared to the gentamicin-only group, though not all differences were statistically significant [39].

Aksoy et al. assigned rats into four groups, and animals received 40 mg/kg TQ daily for 14 days by oral gavage. There were significant differences between the DPOAE amplitudes of the amikacin-only group and the amikacin + TQ group. There were also significant differences between the ABR thresholds; however, exact numerical values were not provided, and results were presented only graphically. Oxidative stress index, determined from blood samples, was significantly higher in the amikacin-only group [40].

The in vitro experimental model using cell cultures obtained in the form of micro-explants of the organ of Corti from mice evaluated the protective effects of TQ at different concentrations (10, 100, and 1000 µM) against gentamicin-induced ototoxicity. Fluorescence microscopy was performed on each day for 3 days, and survival curves were generated. TQ at the lowest concentration was associated with higher hair cell survival at 24 h compared to the gentamicin-only group. This difference was not observed at higher concentrations or at later time points (48–72 h) [41].

#### 3.4.2. Noise-Induced Hearing Loss

Aksoy et al. exposed rats to 105 dB white noise for 4 h. 10 mg/kg i.p. TQ was administered 24 h before the acoustic trauma and continued for 10 days after exposure. DPOAE measurements for both groups were presented graphically. The acoustic trauma + TQ group showed higher response amplitudes than the acoustic trauma-only group at several frequencies; however, no exact numerical values or inter-group statistical results were provided. A statistically significant difference was detected between the ABR threshold of the two groups by day 10 after exposure to acoustic trauma (41.8 ± 4.8 vs. 31.2 ± 5.0) [42].

A similar model was used by Ogurlu et al., exposing rats to 120 dB white noise for 4 h. One group received 20, the other 40 mg/kg i.p. TQ, both administered 30 min before noise exposure, then 24 and 48 h after. Both treatment groups showed significantly higher post-treatment DPOAE values at 2800, 4000, and 6000 Hz, with the low-dose group also showing higher values at 2800 Hz. Post-treatment ABR thresholds were significantly lower than post-trauma values; in the low-dose group, they did not differ from pre-trauma values, while in the high-dose group, they remained significantly higher. At 96 h, ABR and DPOAE differed significantly between treatment and control groups, but no differences were observed immediately after exposure. Exact numerical values were not provided, as results were presented only graphically [43].

The most recent study, conducted by Tekin et al., exposed rats to 100 dB white noise for 16 h, and 10 mg/kg TQ was administered i.p. every day for 2 days before and 8 days after noise exposure. The acoustic trauma group showed a significant decrease in signal-to-noise ratio values on day 1 post-trauma (25–30 dB pre-trauma to 5–10 dB), with partial recovery thereafter. In contrast, the thymoquinone + trauma group did not show a significant reduction in DPOAE on day 1 or subsequent measurements. The thymoquinone-only group showed no significant changes over time [44].

Culhaoglu et al. administered 2 mL/kg NSO orally for 3 days after exposing rats to 107 dB white noise. Post-treatment ABR thresholds were significantly different between the no-treatment and treatment groups (49 ± 10.2 vs. 42 ± 8.9) [45].

#### 3.4.3. Age-Related Hearing Loss

C57/BL/6J mice, aged 8 months, were administered TQ i.p.: 20 mg/kg or 40 mg/kg, 3 times/week for one month. Behavioral assessment of hearing was performed 24 h after the last dose of TQ, and results showed a significant difference between control and treatment groups in the conditioned avoidance behavioral test. Compared with the thresholds of the control group (20–24), the thresholds of the low-dose TQ-treatment group were significantly decreased at 16 kHz (12–15). Furthermore, the average thresholds obtained from the high-dose group were significantly lower at all frequencies than those of the control group (8–10). Histological analysis of the inner ear revealed a lower degree of inner ear damage, especially in the high-dose group [46].

All the results of the individual clinical trials and experimental studies that were included in our review are synthesized and presented in Table 1.

### 3.5. Quantitative Synthesis

Overall Effect

**Figure 3:**
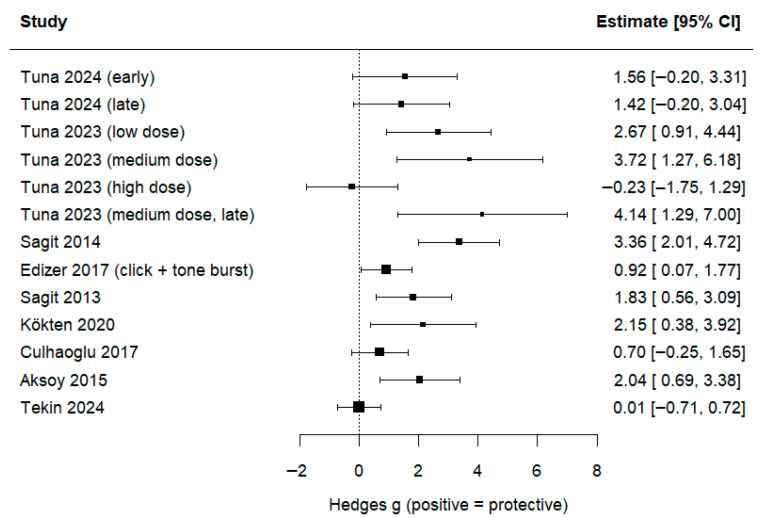
Overall random-effects meta-analysis of the protective effect of NSO/TQ (multi-arm studies are displayed as separate comparisons) [34,35,36,37,38,39,42,44,45].

Across 9 studies, 14 comparisons were found eligible for quantitative synthesis. The REML demonstrated a large and statistically robust protective effect of NSO/TQ across ototoxicity models (Hedges’ g = 1.57; 95% CI: 0.93–2.21; *p* < 0.0001) (Figure 3). Between-study heterogeneity was moderate–high (τ^2^ = 0.92; I^2^ = 66.4%). The 95% prediction interval ranged from –0.41 to 3.55, indicating that while the average effect is strongly protective, individual future studies may yield smaller or non-significant effects due to expected variability across experimental models.

To account for the dependence introduced by multi-arm experimental designs, a multilevel random-effects model with a study-level random intercept was fitted. The pooled effect remained significant (g = 1.35; 95% CI: 0.64–2.06; *p* = 0.0002), confirming that the overall effect was not inflated by clustering of multiple arms within the same study.

Subgroup Analyses

The treatment effect differed significantly according to the mechanism of cochlear injury. Subgroup results are summarized in Table 2.

Both compounds showed statistically significant positive effect sizes (Table 3). Their confidence intervals overlapped, indicating no statistically detectable difference between NSO and TQ.

Outcome measure significantly moderated treatment effects (Table 4). ABR thresholds consistently identified strong protective effects, whereas DPOAE outcomes were highly variable and did not show a consistent treatment benefit.

Publication Bias and Sensitivity Tests

**Figure 4:**
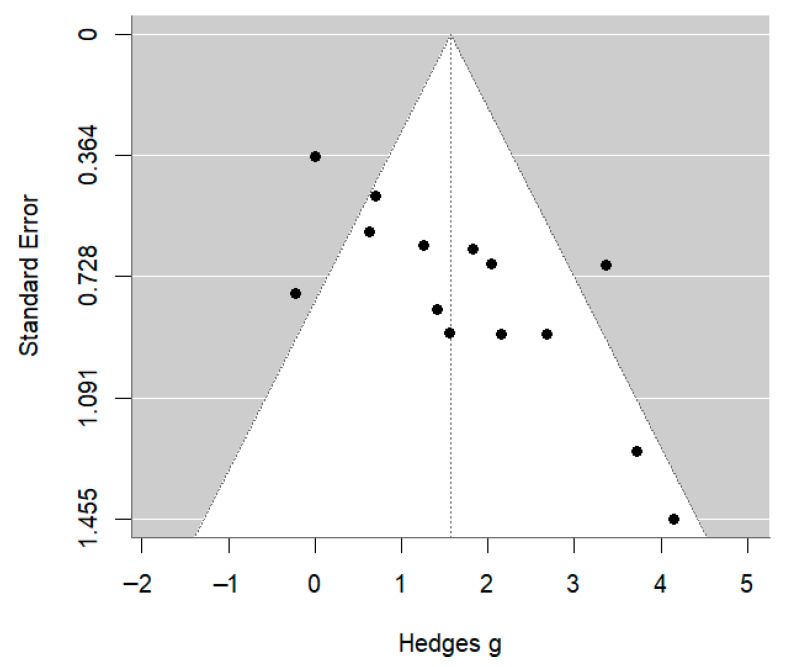
Funnel plot of effect sizes assessing small-study effects.

Visual inspection of the funnel plot suggested asymmetry (Figure 4), confirmed by Egger’s regression (t = 3.89; *p* = 0.0021). This pattern likely reflects small-study effects and true methodological heterogeneity, which are common in preclinical research.

Leave-one-out sensitivity analysis demonstrated that no single comparison materially altered the pooled effect (range: g = 1.37–1.72), and heterogeneity remained moderate (I^2^ 55–70%). Influence diagnostics identified no outlying or disproportionately influential studies.

## 4. Discussion

Our systematic review set out to rigorously evaluate the potential protective role of NS and its active constituent, TQ, in the prevention of acquired SNHL. The evidence gathered provides compelling preclinical evidence that NS can protect the inner ear from various types of damage. However, to date, no clinical evidence supports these data.

Principal findings

Across the included animal studies, NS consistently preserved hearing function, with a notable magnitude of protection—in some cases, completely preventing hearing loss. These findings were further supported by our quantitative synthesis. In the subset of studies with extractable numerical data, the REML demonstrated a large and statistically significant pooled protective effect (Hedges’ g = 1.57, 95% CI 0.93–2.21, *p* < 0.0001). A multilevel model accounting for multiple experimental arms yielded a similarly strong effect (g = 1.35, 95% CI 0.64–2.06), confirming that the findings were not inflated by clustered study designs.

Subgroup analyses provided further insight into injury-specific efficacy. NSO/TQ significantly attenuated cisplatin- and gentamicin-induced ototoxicity, while the effect on noise-induced hearing loss showed a directional but non-significant trend. Both NSO and TQ yielded comparable effect sizes, with overlapping confidence intervals. Outcome-specific analyses revealed that ABR thresholds consistently detected protective effects, whereas DPOAE SNR measures were highly variable.

These quantitative results are consistent with the narrative evidence. For example, TQ administration fully protected against aminoglycoside-induced hearing loss [40]. In addition, several studies demonstrated the protective effects of NS against cisplatin-induced hearing loss. Pretreatment with NSO or TQ significantly preserved OHC integrity, maintained ABR response thresholds. These findings collectively support the hypothesis that the antioxidant and anti-inflammatory properties of NS confer substantial protection against cisplatin-induced cochlear damage in preclinical models [34,35]. Another study showed that NSO facilitated significant recovery following acoustic trauma [45]. These findings are noteworthy given the robustness of the ototoxicity models employed, which reliably induce hearing loss.

Both forms, NSO and TQ, through their antioxidant and anti-apoptotic actions, provided significant protection in all identified studies against ototoxicity. Hearing thresholds were preserved completely or partially with NSO/TQ treatment. Secondary outcomes such as cochlear histopathology and oxidative stress markers also showed statistically significant differences. NSO/TQ consistently improved hearing outcomes following acoustic trauma as well. Immediate noise damage still occurs, especially at very high noise levels, but NS-treated subjects demonstrated significant hearing recovery over subsequent days.

Biological plausibility

The mechanisms by which NS exerts its protective effects appear to be multifactorial. Most prominently, NS mitigates oxidative stress, a key pathological process in SNHL. Due to its high metabolic activity and poor regenerative capacity, the cochlea is highly susceptible to damage mediated by oxidative stress. Most causes of acquired SNHL converge on the generation of ROS, which forms the basis of pathogenesis that further leads to lipid peroxidation, DNA damage, and ultimately, sensory hair cell death [47]. The stronger effects seen in cisplatin- and gentamicin-induced ototoxicity compared with noise-induced trauma during quantitative synthesis are biologically plausible, as these drug models are dominated by oxidative and inflammatory injury pathways directly targeted by NSO/TQ, whereas noise trauma includes a substantial mechanical component that is intrinsically less responsive to antioxidant therapy.

Different antioxidants and anti-inflammatories have long been investigated as otoprotective agents. What stands out about NS is its potency and multimodal mechanism of action. TQ, its active constituent, is a lipophilic quinone that can penetrate tissues and hunt free radicals. Moreover, it modulates antioxidant enzymes and increases the production of endogenous antioxidants [30,48]. Additionally, NS exhibits anti-inflammatory effects through inhibition of pro-inflammatory cytokines, which can reduce secondary damage after the initial insult [49,50]. The results of this review strongly support the translation of these mechanisms into preserving both structure and function of the inner ear.

The in vitro experimental model found minimal protective effect of TQ against gentamicin-induced toxicity. This limited effect in vitro may suggest that the protective mechanism of TQ is not primarily mediated through direct action on sensory hair cells, but rather involves interactions with adjacent cochlear or inner ear structures, which are not fully represented in isolated cell culture systems [41].

Timing and Dosing

A nuanced point in the literature is the timing of NS administration, as timing emerged as a critical determinant of efficacy. Our results showed that initiating NS/TQ before or at the time of the insult offers the best chance to preserve hearing. This implies that NS could be both prophylactic and therapeutic, but its role differs; it can preserve hearing function through the insult or, therapeutically, rescue hearing if administered early [51,52]. In clinical translation, these results suggest that NS could be given prior to high-risk events—e.g., before cisplatin chemotherapy—or immediately after an unexpected insult, such as a sudden noise exposure. Timing is crucial: starting as early as possible yields the best results, since once hair cells are lost, they cannot regenerate. Nevertheless, partially injured hair cells may be saved by limiting apoptosis, which is what NS/TQ appear to do.

With respect to dosing, both intraperitoneal and oral regimens demonstrated protective effects in animal models. For intraperitoneal administration, TQ was typically administered at 10–40 mg/kg in rodents, with consistent otoprotective effects [34,36,42,43,44,46]. Intraperitoneal NSO showed robust protection at 0.3 mL/kg, while 0.1 mL/kg or 3 mL/kg did not show any statistically significant protection [37,38]. Oral administration in animals also showed protective effects; doses of 2–3 mL/kg NSO conferred significant protection against NIHL and cisplatin ototoxicity [35,45]. One study demonstrated a significant protective effect of orally administered TQ against gentamicin-induced ototoxicity at a dose of 40 mg/kg [40].

Safety

An encouraging finding of this review is that both NSO and TQ appear to be safe and well-tolerated. In rodent models, even high doses of TQ (40 mg/kg) and NSO (2–3 mL/kg) administered systemically did not cause any organ damage. Some studies suggest extremely high doses of TQ (2–3 g/kg) or NSO (25 mL/kg) may carry hepatic or renal toxicity. However, otoprotective effects were observed within a reasonably safe range [53,54]. In a human trial, 1 g/day of NSO orally did not cause any reported side effects [55]. These findings align with human studies for other uses of NS, where typical doses of 1–3 g/day resulted in no or minimal adverse events, such as mild gastrointestinal upsets [56]. Safety profile is important, given that NS would likely be used in a preventive setting in otherwise healthy individuals, its excellent risk–benefit profile is particularly encouraging.

Clinical evidence

A single human randomized controlled trial (RCT), investigating NSO’s protective effects in patients with Meniere’s disease, was identified during our search but was excluded from the synthesis due to methodological differences from the preclinical studies. Patients received 1 g of oil orally once daily for three months, and then hearing was evaluated using pure-tone audiometry, while tinnitus and vertigo were assessed through standardized questionnaires (tinnitus handicap inventory questionnaire and dizziness handicap inventory questionnaire). The study did not show significant improvement, likely reflecting the complex pathophysiology of the disease, the small sample size, and the short trial duration. Nevertheless, this study underscores both the translational interest in NS and the current lack of robust human data.

Limitations

Despite these promising findings, several limitations must be acknowledged. The reviewed animal studies were heterogeneous in design, involving different species, ototoxic models, dosing regimens, routes of administration, and outcome measures. While this heterogeneity complicates direct comparisons, it justifies the use of random-effects and multilevel models in the quantitative synthesis. Notably, although heterogeneity was moderate–high (I^2^ = 66%), the protective effect remained significant across all sensitivity analyses, including leave-one-out recalculations and influence diagnostics. While it shows the versatility of NS in preventing SNHL, it makes comparison and drawing objective conclusions more difficult. Also, as discussed, some of the animal studies had incomplete reporting methods, yet the effects were large enough that—even accounting for potential bias—a genuine protective effect remains likely. Egger’s regression indicated funnel plot asymmetry, which may reflect small-study effects typical of preclinical research rather than true publication bias. Regarding the review process, the exclusion of non-English studies and gray literature may have introduced selection bias. Nonetheless, we are confident that these methodological limitations did not impact the overall results. Another limitation is the lack of prospective protocol registration; although written beforehand, the protocol was only retrospectively registered on OSF for transparency (DOI: 10.17605/OSF.IO/M49UF).

Implications and Future Research

Comparisons with currently used otoprotective strategies further emphasize the relevance of NS. Corticosteroids, such as dexamethasone, are commonly used to treat sudden SNHL and have also been studied in noise- and drug-induced hearing loss due to their potent anti-inflammatory effects. However, they have numerous side effects, particularly when administered orally [13,57]. Different antioxidants, such as amifostine and N-acetylcysteine, have shown mixed outcomes and pose concerns related to possible side effects and interference with chemotherapeutic efficacy [58]. TQ may have an edge, as it not only avoids attenuating cisplatin’s chemotherapeutic effect but may even enhance it, through synergistic pro-apoptotic effects in malignant cells [59].

To guide future translational research, several methodological considerations emerge from the current evidence. First, dosing strategies should be informed by the high variability in animal studies, with future work focusing on defining pharmacokinetically justified dosing ranges rather than arbitrary mg/kg equivalence. Second, formulation purity (cold-pressed NSO vs. standardized TQ extract) should be explicitly reported, as variability in phytochemical content may meaningfully influence efficacy. Third, routes of administration warrant systematic comparison, as most existing studies used systemic delivery, whereas topical or trans-tympanic administration may offer higher cochlear exposure with fewer systemic risks. Future preclinical studies should incorporate standardized outcome measures, controlled timing of administration, and blinding to reduce bias and improve comparability. Comparative studies with standard therapies such as dexamethasone, as well as combination regimens, should be explored.

## 5. Conclusions

Our systematic review highlights NS and its active compound TQ as promising otoprotective agents against acquired SNHL. Quantitative synthesis demonstrates that Nigella sativa and thymoquinone exert a measurable protective effect against acquired sensorineural hearing loss in preclinical models, reflected by reduced ABR threshold shifts and improved DPOAE amplitudes.

While no clinical evidence currently confirms these benefits, the preclinical data provide a compelling rationale for translation into human studies. These findings support the potential of NSO/TQ as otoprotective agents, but further standardized, high-quality studies are needed to strengthen the evidence base and guide future translation into clinical contexts. Given its favorable safety profile, accessibility, and potential cost-effectiveness, NS could become a valuable preventive or adjunctive therapy for high-risk populations—such as patients undergoing platinum-based chemotherapy or individuals exposed to occupational noise.

## Figures and Tables

**Table 1:** Synthesis of all included studies. [34,35,36,37,38,39,40,41,42,43,44,45,46].

Authors (Year)	Experimental Model (Species/Strain)	Sample Size (Per Group)	Hearing Loss Model	Intervention	Comparator/Control	Route and Dose	Timing	Outcomes Measured	Results (Protective Effect)
**Ototoxicity models**									
Tuna & Tüzemen 2024 [38]	In vivo experimental Sprague Dawley rats	6	Gentamicin 120 mg/kg/day i.p. for 10 days	NSO	Saline	Intraperitoneal 0.3 mL/kg/day daily for 5 days	After exposure	ABR	++
Tuna & Tüzemen 2023 [37]	In vivo experimental Sprague Dawley rats	8	Gentamicin 120 mg/kg/day i.p. for 10 days	NSO	Saline	Intraperitoneal 0.1 mL/kg/day daily for 5 days 0.3 mL/kg/day daily for 5 days 3 mL/kg/day daily for 5 days	Pre-exposure & after	ABR	++
Kökten et al., 2020 [35]	In vivo experimental Wistar albino rats	6	Cisplatin 5 mg/kg i.p. on days 1, 3, and 5	NSO	Saline	Oral 3 mL/day on days 1, 3, 5, 7, 9	Concurrently	ABR Histopathology	+
Edizer et al., 2017 [39]	In vivo experimental Dunkin-Hartley albino guinea pigs	7	Gentamicin 100 mg/kg/day per day i.p. for 3 weeks	NSO	Saline	Intratympanic 4 mL/kg divided into equal doses once a week for 3 weeks	Concurrently	ABR Histopathology	+
Aksoy et al., 2015 [40]	In vivo experimental Wistar albino rats	8	Amikacin 600 mg/kg/day intramuscularly for 14 days	TQ	Negative control	Oral 40 mg/kg/day daily for 14 days	Concurrently	DPOAE ABR	++
Sagit et al., 2014 [36]	In vivo experimental Sprague Dawley rats	12	Gentamicin 120 mg/kg/day i.p. for 15 days	TQ	Corn oil	Intraperitoneal 20 mg/kg/day daily for 15 days	Concurrently	ABR Histopathology Immunohistochemestry	++
Sagit et al., 2013 [34]	In vivo experimental Sprague Dawley rats	10	Cisplatin 15 mg/kg i.p. 1 day	TQ	Negative control	Intraperitoneal 40 mg/kg/day daily for 5 days	Pre-exposure & after	DPOAE ABR	++
Noack et al., 2017 [41]	In vitro experimental Mouse cochlear hair cells		Gentamicin 200 µM direct application	TQ	Negative control	Direct application 10 µM, 100 µM, 1000 µM	Pre-exposure	GFP fluorescence microscopy Hair cell survival rate	+
**Acoustic trauma models**									
Tekin et al., 2024 [44]	In vivo experimental Wistar albino rats	8	100 dB white noise at a frequency of 4 kHz for 16 h	TQ	Negative control	Intraperitoneal 10 mg/kg/day daily for 10 days	Pre-exposure & after	DPOAE	++
Culhaoglu et al., 2017 [45]	In vivo experimental Sprague Dawley rats	10	107 dB white noise at a frequency of 4 kHz for 12 h	NSO	Negative control	Oral 2 mL/kg/day daily for 3 days	After exposure	ABR	++
Ogurlu et al., 2017 [43]	In vivo experimental Sprague Dawley rats	8	120 dB white noise at a frequency of 4 kHz for 4 h	TQ	Corn oil	Intraperitoneal 20 mg/kg/day daily for 3 days 40 mg/kg/day daily for 3 days	Pre-exposure & after	DPOAE ABR	++
Aksoy et al., 2015 (acoustic trauma) [42]	In vivo experimental Wistar albino rats	8	105 dB white noise at a frequency of 4 kHz for 4 h	TQ	Negative control	Intraperitoneal 10 mg/kg/day daily for 11 days	Pre-exposure & after	DPOAE ABR	++
**Age-related hearing loss model**									
Salam et al., 2021 [46]	In vivo experimental Black 6 mice (C57BL/6J)	6		TQ	Corn oil	Intraperitoneal 20 mg/kg/day 40 mg/kg/day 3 days/week for 1 month		Behavioral Audiogram Histopathology	++

Nigella sativa oil (NSO), thymoquinone (TQ), acoustic brainstem response (ABR), distorted product otoacoustic emissions (DPOAE); protective effect increased + slightly (not statistically significant)/++ strongly (statistically significant) or – no effect.

**Table 2:** Synthesis of all included studies.

Model	g	95% CI	*p*-Value
Cisplatin	**1.97**	0.28–3.65	0.022
Gentamicin	**1.82**	0.99–2.65	<0.0001
Noise	0.82	−0.40–2.03	0.188

Effect sizes expressed as Hedges’ g. Positive values indicate a protective effect. Differences between subgroups were statistically significant according to the omnibus test for moderators (QM < 0.0001).

**Table 3:** Subgroup analysis comparing Nigella sativa oil (NSO) with thymoquinone (TQ).

Compound	g	95% CI	*p*-Value
NSO	**1.53**	0.71–2.35	0.0003
TQ	**1.69**	0.53–2.85	0.0044

Effect sizes expressed as Hedges’ g. Positive values indicate a protective effect. Differences between subgroups were statistically significant according to the omnibus test for moderators (QM < 0.0001).

**Table 4:** Overall random-effects meta-analysis of the protective effect of NSO/TQ (multi-arm studies are displayed as separate comparisons).

Outcome	g	95% CI	*p*-Value
ABR	**1.72**	1.10–2.34	<0.0001
DPOAE	0.01	−1.75–1.76	0.992

Effect sizes expressed as Hedges’ g. Positive values indicate a protective effect. Differences between subgroups were statistically significant according to the omnibus test for moderators (QM < 0.0001).

## Data Availability

The original extensive data extraction spreadsheet presented in the study is openly available.

## Supplementary Materials

The following supporting information can be downloaded at: https://www.mdpi.com/article/10.3390/jcm14238433/s1, Table S1: meta_full_ready; Table S2: Nigella_Sativa_Hearing_Loss_Data_Extraction; File S1: PRISMA_2020_checklist; File S2: Systematic_Review_Protocol_Revised.

## Abbreviations

The following abbreviations are used in this manuscript:

SNHL	Sensorineural hearing loss
NS	Nigella sativa
TQ	Thymoquinone
PRISMA	Preferred Reporting Items for Systematic Reviews and Meta-analyses
SYRCLE	Systematic Review Center for Laboratory Animal Experiments
ROS	Reactive oxygen species
NIHL	Noise-induced hearing loss
AIHL	Age-related hearing loss
ABR	Auditory brainstem response
DPOAE	Distortion product otoacoustic emission
PTA	Pure-tone audiometry
RCT	Randomized controlled trial
NSO	Nigella sativa oil

## Appendix A

**Table A1:** T Medical Subject Headings terms used for generating the search string.

(“Nigella sativa” OR “Nigella sativa oil” OR “black cumin” OR “black seed” OR “Thymoquinone”) AND (“hearing loss” OR “sensorineural hearing loss” OR “deafness” OR “ototoxicity” OR “otoprotection” OR “auditory protection” OR “hearing protection” OR “acoustic trauma” OR “noise-induced hearing loss” OR “ototoxic hearing loss” OR “cochlear damage” OR “auditory threshold”)

## References

[1] World Health Organization (2021). World Report on Hearing.

[2] Plontke S.K., Meisner C., Agrawal S., Cayé-Thomasen P., Galbraith K., Mikulec A.A., Parnes L., Premakumar Y., Reiber J., Schilder A.G.M. (2022). Intratympanic Corticosteroids for Sudden Sensorineural Hearing Loss. Cochrane Database Syst. Rev..

[3] Crundwell G., Gomersall P., Baguley D.M. (2016). Ototoxicity (Cochleotoxicity) Classifications: A Review. Int. J. Audiol..

[4] Dillard L.K., Lopez-Perez L., Martinez R.X., Fullerton A.M., Chadha S., McMahon C.M. (2022). Global Burden of Ototoxic Hearing Loss Associated with Platinum-Based Cancer Treatment: A Systematic Review and Meta-Analysis. Cancer Epidemiol..

[5] Dasari S., Bernard Tchounwou P. (2014). Cisplatin in Cancer Therapy: Molecular Mechanisms of Action. Eur. J. Pharmacol..

[6] Marshak T., Steiner M., Kaminer M., Levy L., Shupak A. (2014). Prevention of Cisplatin-Induced Hearing Loss by Intratympanic Dexamethasone: A Randomized Controlled Study. Otolaryngol.-Head Neck Surg..

[7] Li J., Woo C.W. (2016). Finding Ways to Solve or Prevent Aminoglycoside-Induced Ototoxicity?. Ann. Transl. Med..

[8] Natarajan N., Batts S., Stankovic K.M. (2023). Noise-Induced Hearing Loss. J. Clin. Med..

[9] Agrawal Y., Platz E.A., Niparko J.K. (2008). Prevalence of Hearing Loss and Differences by Demographic Characteristics Among US Adults Data from the National Health and Nutrition Examination Survey, 1999–2004. Arch. Intern. Med..

[10] Yuan C., Ma T., Liu M., Zeng X., Tang G., Xing Y., Zhang T. (2024). Ferroptosis, Oxidative Stress and Hearing Loss: Mechanistic Insights and Therapeutic Opportunities. Heliyon.

[11] Maniaci A., La Via L., Lechien J.R., Sangiorgio G., Iannella G., Magliulo G., Pace A., Mat Q., Lavalle S., Lentini M. (2024). Hearing Loss and Oxidative Stress: A Comprehensive Review. Antioxidants.

[12] Teraoka M., Hato N., Inufusa H., You F. (2024). Role of Oxidative Stress in Sensorineural Hearing Loss. Int. J. Mol. Sci..

[13] Lavigne P., Lavigne F., Saliba I. (2016). Intratympanic Corticosteroids Injections: A Systematic Review of Literature. Eur. Arch. Oto-Rhino-Laryngol..

[14] Daldal A., Odabasi O., Serbetcioglu B. (2007). The Protective Effect of Intratympanic Dexamethasone on Cisplatin-Induced Ototoxicity in Guinea Pigs. Otolaryngol.-Head Neck Surg..

[15] Haake S.M., Dinh C.T., Chen S., Eshraghi A.A., Van De Water T.R. (2009). Dexamethasone Protects Auditory Hair Cells against TNFα-Initiated Apoptosis via Activation of PI3K/Akt and NFκB Signaling. Hear. Res..

[16] Li G., Yang H., Zhang P., Guo Y., Yuan L., Xu S., Yuan Y., Xiong H., Yin H. (2024). Insights into the Molecular Underlying Mechanisms and Therapeutic Potential of Endoplasmic Reticulum Stress in Sensorineural Hearing Loss. Front. Mol. Neurosci..

[17] Freyer D.R., Chen L., Krailo M.D., Knight K., Villaluna D., Bliss B., Pollock B.H., Ramdas J., Lange B., Van Hoff D. (2017). Effects of Sodium Thiosulfate versus Observation on Development of Cisplatin-Induced Hearing Loss in Children with Cancer (ACCL0431): A Multicentre, Randomised, Controlled, Open-Label, Phase 3 Trial. Lancet Oncol..

[18] Hazlitt R.A., Min J., Zuo J. (2018). Progress in the Development of Preventative Drugs for Cisplatin-Induced Hearing Loss. J. Med. Chem..

[19] Yu D., Gu J., Chen Y., Kang W., Wang X., Wu H. (2020). Current Strategies to Combat Cisplatin-Induced Ototoxicity. Front. Pharmacol..

[20] Brock P., Meijer A., Kogner P., Ansari M., Capra M., Geller J., Heuvel-Eibrink M.V.D., Knight K., Kruger M., Lindemulder S. (2023). Sodium Thiosulfate as Cisplatin Otoprotectant in Children: The Challenge of When to Use It. Pediatr. Blood Cancer.

[21] Meijer A.J.M., Diepstraten F.A., Ansari M., Bouffet E., Bleyer A., Fresneau B., Geller J.I., Huitema A.D.R., Kogner P., Maibach R. (2024). Use of Sodium Thiosulfate as an Otoprotectant in Patients with Cancer Treated With Platinum Compounds: A Review of the Literature. J. Clin. Oncol..

[22] Ahmad A., Husain A., Mujeeb M., Khan S.A., Najmi A.K., Siddique N.A., Damanhouri Z.A., Anwar F. (2013). A Review on Therapeutic Potential of *Nigella sativa*: A Miracle Herb. Asian Pac. J. Trop. Biomed..

[23] Alberts A., Moldoveanu E.T., Niculescu A.G., Grumezescu A.M. (2024). *Nigella sativa*: A Comprehensive Review of Its Therapeutic Potential, Pharmacological Properties, and Clinical Applications. Int. J. Mol. Sci..

[24] Arshad M.T., Maqsood S., Ikram A., Abdullahi M.A. (2025). Functional, Nutraceutical, and Pharmacological Properties of Black Seed. Food Sci. Nutr..

[25] Pandey R., Pandey B., Bhargava A. (2025). An Updated Review on the Phytochemistry and Pharmacological Activity of Black Cumin (*Nigella sativa* L.). Adv. Chin. Med..

[26] Hamdy N.M., Taha R.A. (2009). Effects of *Nigella sativa* Oil and Thymoquinone on Oxidative Stress and Neuropathy in Streptozotocin-Induced Diabetic Rats. Pharmacology.

[27] Hendriyanto D., Helmi H. (2023). Protective Role of *Nigella sativa* Oil against Cisplatin-Induced Ototoxicity: A Literature Review. J. Med. Sci. (Berk. Ilmu Kedokt.).

[28] Nainawat K.S., Budakoti A., Kumari N., Verma R.S., Gupta A. (2025). Pharmaceutical Perspectives of Thymoquinone, a Lead Molecule from Black Cumin (*Nigella sativa* L.) with Diverse Biological Targets. Food Chem. Adv..

[29] Isaev N.K., Genrikhs E.E., Stelmashook E.V. (2023). Antioxidant Thymoquinone and Its Potential in the Treatment of Neurological Diseases. Antioxidants.

[30] Nader M.A., El-Agamy D.S., Suddek G.M. (2010). Protective Effects of Propolis and Thymoquinone on Development of Atherosclerosis in Cholesterol-Fed Rabbits. Arch. Pharm. Res..

[31] Page M.J., McKenzie J.E., Bossuyt P.M., Boutron I., Hoffmann T.C., Mulrow C.D., Shamseer L., Tetzlaff J.M., Akl E.A., Brennan S.E. (2021). The PRISMA 2020 Statement: An Updated Guideline for Reporting Systematic Reviews. BMJ.

[32] Sterne J.A.C., Savović J., Page M.J., Elbers R.G., Blencowe N.S., Boutron I., Cates C.J., Cheng H.Y., Corbett M.S., Eldridge S.M. (2019). RoB 2: A Revised Tool for Assessing Risk of Bias in Randomised Trials. BMJ.

[33] Hooijmans C.R., Rovers M.M., De Vries R.B.M., Leenaars M., Ritskes-Hoitinga M., Langendam M.W. (2014). SYRCLE’s Risk of Bias Tool for Animal Studies. BMC Med. Res. Methodol..

[34] Sagit M., Korkmaz F., Akcadag A., Somdas M.A. (2013). Protective Effect of Thymoquinone against Cisplatin-Induced Ototoxicity. Eur. Arch. Oto-Rhino-Laryngol..

[35] Kökten N., Eğilmez O.K., Erinç M., Işın Doğan Ekici A., Şerifler S., Yeşilada E., Kalcıoğlu M.T. (2020). The Protective Effect of *Nigella sativa* Oil against Experimentally Induced Cisplatin Ototoxicity: An Animal Study. J. Int. Adv. Otol..

[36] Sagit M., Korkmaz F., Gürgen S.G., Kaya M., Akcadag A., Ozcan I. (2014). The Protective Role of Thymoquinone in the Prevention of Gentamicin Ototoxicity. Am. J. Otolaryngol.-Head Neck Med. Surg..

[37] Tuna B., Tüzemen G. (2023). Protective Effects of *Nigella sativa* Oil against Gentamicin-Induced Ototoxicity in Rats: A Dose-Ranging Study. Int. J. Pediatr. Otorhinolaryngol..

[38] Tuna B., Tüzemen G. (2024). Is Gentamicin-Induced Ototoxicity Reversible with Delayed Administration of *Nigella sativa* Oil? An Experimental Study. Turk. J. Ear Nose Throat.

[39] Edizer D.T., Yigit O., Cinar Z., Gul M., Kara E., Yigitcan B., Hayır D., Atas A. (2017). Protective Role of Intratympanic *Nigella sativa* Oil against Gentamicin Induced Hearing Loss. Int. J. Pediatr. Otorhinolaryngol..

[40] Aksoy F., Dogan R., Ozturan O., Tugrul S., Veyseller B., Ozer O.F., Pektas A. (2015). An Evaluation of the Protective Effects of Thymoquinone on Amikacin-Induced Ototoxicity in Rats. Clin. Exp. Otorhinolaryngol..

[41] Noack V., Pak K., Jalota R., Kurabi A., Ryan A.F. (2017). An Antioxidant Screen Identifies Candidates for Protection of Cochlear Hair Cells from Gentamicin Toxicity. Front. Cell Neurosci..

[42] Aksoy F., Dogan R., Yenigun A., Veyseller B., Ozturan O., Ozturk B. (2015). Thymoquinone Treatment for Inner-Ear Acoustic Trauma in Rats. J. Laryngol. Otol..

[43] Ogurlu M., Celebi Erdivanli O., Tumkaya L., Ozgur A., Ozergin Coskun Z., Terzi S., Demirci M., Dursun E. (2017). The Therapeutic Effect of Thymoquinone on Acoustic Trauma-Induced Hearing Loss in Rats. Eur. Arch. Oto-Rhino-Laryngol..

[44] Tekin M.S., Ayçiçek A., Bucak A., Ulu Ş., Okur E. (2024). The Effect of Thymoquinone on Acoustic Trauma-Induced Hearing Loss in Rats. Cureus.

[45] Culhaoglu B., Erbek S.S., Erbek S., Hizal E. (2017). Protective Effect of *Nigella sativa* Oil on Acoustic Trauma Induced Hearing Loss in Rats. Audiol. Res..

[46] Salam S.A., Mostafa F., Alnamshan M.M., Elshewemi S.S., Sorour J.M. (2021). Thymoquinone Ameliorates Age-Related Hearing Loss in C57BL/6J Mice by Modulating Sirt1 Activity and Bak1 Expression. Biomed. Pharmacother..

[47] Kamogashira T., Fujimoto C., Yamasoba T. (2015). Reactive Oxygen Species, Apoptosis, and Mitochondrial Dysfunction in Hearing Loss. BioMed Res. Int..

[48] Ali B.H., Blunden G. (2003). Pharmacological and Toxicological Properties of *Nigella sativa*. Phytother. Res..

[49] Torequl Islam M. (2016). Biological activities and therapeutic promises of *Nigella sativa* L.. Int. J. Pharma Sci. Sci. Res..

[50] Ansari Z., Nasiruddin M., Khan R., Haque S. (2017). Protective Role of *Nigella sativa* in Diabetic Nephropathy: A Randomized Clinical Trial. Saudi J. Kidney Dis. Transplant..

[51] Gentilin E., Simoni E., Candito M., Cazzador D., Astolfi L. (2019). Cisplatin-Induced Ototoxicity: Updates on Molecular Targets. Trends Mol. Med..

[52] El Sabbagh N.G., Sewitch M.J., Bezdjian A., Daniel S.J. (2017). Intratympanic Dexamethasone in Sudden Sensorineural Hearing Loss: A Systematic Review and Meta-Analysis. Laryngoscope.

[53] Badary O.A., Al-Shabanah O.A., Nagi M.N., Al-Bekairi A.M., Elmazar M.M.A. (1998). Acute and Subchronic Toxicity of Thymoquinone in Mice. Drug Dev. Res..

[54] Gholamnezhad Z., Havakhah S., Boskabady M.H. (2016). Preclinical and Clinical Effects of *Nigella sativa* and Its Constituent, Thymoquinone: A Review. J. Ethnopharmacol..

[55] Motesadi Zarandi M., Rabbani Z., Rabbani Anari M., Kouhi A., Zeinaloo M. (2023). A Study of Efficacy of *Nigella sativa* in Treatment of Meniere’s Disease: A Randomized, Placebo Controlled Clinical Trial. J. Otol..

[56] Jarmakiewicz-Czaja S., Zielińska M., Helma K., Sokal A., Filip R. (2023). Effect of *Nigella sativa* on Selected Gastrointestinal Diseases. Curr. Issues Mol. Biol..

[57] Ekborn A., Hansson J., Ehrsson H., Eksborg S., Wallin I., Wagenius G., Laurell G. (2004). High-Dose Cisplatin with Amifostine: Ototoxicity and Pharmacokinetics. Laryngoscope.

[58] Peng L., Liu A., Shen Y., Xu H.Z., Yang S.Z., Ying X.Z., Liao W., Liu H.X., Lin Z.Q., Chen Q.Y. (2013). Antitumor and Anti-Angiogenesis Effects of Thymoquinone on Osteosarcoma through the NF-ΚB Pathway. Oncol. Rep..

[59] Zaghlol D.A.A., Kamel E.S., Mohammed D.S., Abbas N.H. (2012). The Possible Toxic Effect of Different Doses of *Nigella sativa* Oil on the Histological Structure of the Liver and Renal Cortex of Adult Male Albino Rats. Egypt. J. Histol..

