# Supplementary material for: Nigella Sativa and Thymoquinone for Prevention or Mitigation of Acquired Sensorineural Hearing Loss: A Systematic Review
Source: J Clin Med. 2025 Nov 27;14(23):8433. doi: 10.3390/jcm14238433 (PMC12693045; doi:10.3390/jcm14238433)
Supplement: Supplementary file 1 [file jcm-14-08433-s001.zip › File S2 Systematic Review Protocol.pdf]

Supplementary Material

Study Protocol: Nigella sativa and Thymoquinone for Prevention or Mitigation of Acquired Sensorineural Hearing Loss – A Systematic Review

1. Administrative Information

Title
Nigella sativa and thymoquinone for prevention or mitigation of acquired sensorineural hearing loss: a systematic review protocol

Registration
The review will not be prospectively registered in PROSPERO, but all protocol details are described here.

Authors and Contributions
Hunor Levente Horvath (guarantor), Violeta Necula, Maximilian George Dindelegan, Cristina Maria Blebea, Victor Esanu, Alma Aurelia Maniu.
All authors contributed to protocol development, search strategy, eligibility criteria, and analysis plan.

2. Rationale

Acquired sensorineural hearing loss (SNHL) is a prevalent and often irreversible condition caused by diverse insults (ototoxic drugs, noise exposure, aging). Oxidative stress and inflammation are common pathological mechanisms. Nigella sativa (NS) and its main bioactive compound, thymoquinone (TQ), possess antioxidant and anti-inflammatory properties and may protect the cochlea from damage. No previous systematic review has synthesized evidence for the protective effects of NS/TQ against acquired SNHL.

3. Objectives

To systematically identify, critically appraise, and synthesize evidence from human, animal, and in-vitro interventional studies evaluating the protective or therapeutic effects of NS or TQ on acquired SNHL.

4. Eligibility Criteria

Population: Human participants of any age with acquired SNHL; experimental animal models; or cochlear cell cultures exposed to ototoxic or traumatic insults.
Intervention: Administration of NS (seed, oil, or extract) or purified TQ by any route (oral, intraperitoneal, intravenous, intratympanic).
Comparator: Placebo, no treatment, or standard therapy.
Outcomes (primary): Quantitative measures of hearing function (Auditory Brainstem Response [ABR], Distortion Product Otoacoustic Emissions [DPOAE], Pure Tone Audiometry [PTA], or equivalent).

Outcomes (secondary): Histological evidence of cochlear protection, biochemical markers of oxidative stress or inflammation, adverse effects or toxicity.
Study design: Randomized controlled trials, controlled laboratory animal studies, and in-vitro experimental models.
Language & timeframe: English, no date restrictions.
Exclusions: Studies focused on conductive hearing loss; non-interventional observational studies; conference abstracts without extractable data; mechanistic studies lacking hearing outcomes.

5. Information Sources

Electronic databases: PubMed/MEDLINE, Web of Science, Scopus, and Embase.
Other sources: Reference lists of included studies and relevant reviews will be screened for additional eligible articles.
Search period: From database inception to the date of final search (1st of March – 23rd of August 2025).

6. Search Strategy

A comprehensive strategy will be designed with controlled vocabulary and free text terms.
Example PubMed syntax:
("Nigella sativa" OR "Nigella sativa oil" OR "black cumin" OR "black seed" OR "Thymoquinone")
AND
("hearing loss" OR "sensorineural hearing loss" OR "deafness" OR "ototoxicity" OR "otoprotection" OR "auditory protection" OR "acoustic trauma" OR "noise-induced hearing loss" OR "cochlear damage" OR "auditory threshold")
Database-specific syntax will be adapted accordingly.

7. Study Records

Data management: Search results will be imported into a reference manager (e.g., Covidence and Mendeley) for deduplication.
Selection process: Two reviewers will independently screen titles/abstracts and full texts against eligibility criteria. Disagreements will be resolved by discussion or a third reviewer.
Data collection process: A standardized extraction form will capture study characteristics, intervention details, comparator details, outcomes and numerical results, risk of bias domains, and adverse events.

8. Outcomes

Primary: Change in quantitative hearing measures (ABR thresholds, DPOAE amplitudes, PTA thresholds).
Secondary: Histological findings of cochlear protection, biochemical markers of oxidative stress or inflammation, and reported adverse effects.

9. Risk of Bias Assessment

Randomized controlled trials: Cochrane Risk of Bias 2 (RoB 2) tool.
Animal studies: SYRCLE risk of bias tool.
Two reviewers will independently assess each domain; disagreements will be resolved by consensus.

10. Data Synthesis

A qualitative narrative synthesis will be performed.
If sufficient homogeneity exists in models and outcome measures, a random-effects meta-analysis will be considered using mean differences for continuous outcomes with 95% confidence intervals.

11. Subgroup and Sensitivity Analyses

If data permit, subgroup analyses will explore: type of model (human vs animal vs in-vitro); type of insult (drug-induced, noise-induced, age-related); intervention type (NS vs TQ); route of administration (systemic vs local).

12. Timeline

Search completion: within two months of protocol finalization
Screening and data extraction: within three months
Data synthesis and manuscript preparation: within six months

13. Funding and Conflicts of Interest

No external funding is planned. Authors declare no conflicts of interest.

Use of This Protocol

This protocol represents the planned methodology established before conducting the review and is provided to satisfy PRISMA-P requirements for transparency and reproducibility.
